# Standardized ileal digestibility of amino acids in corn distillers dried grains with solubles fed to gestating and lactating sows

**DOI:** 10.1093/jas/skaf376

**Published:** 2025-10-30

**Authors:** Ke Wang, Zhao Ma, Min Yang, Lianqiang Che, Shengyu Xu, Yong Zhuo, Bin Feng, Lun Hua, Jian Li, Xuemei Jiang, Zhengfeng Fang, De Wu, Yan Lin

**Affiliations:** Institute of Animal Nutrition, Key Laboratory for Animal Disease-Resistance Nutrition of China, Ministry of Education, Sichuan Agricultural University, Chengdu, Sichuan, 611130, China; School of Bioengineering, Henan University of Technology, Zhengzhou, Henan, 450000, China; Institute of Animal Nutrition, Key Laboratory for Animal Disease-Resistance Nutrition of China, Ministry of Education, Sichuan Agricultural University, Chengdu, Sichuan, 611130, China; Pet Nutrition and Health Research Center, Chengdu Agricultural College, Chengdu, Sichuan, 611130, China; Institute of Animal Nutrition, Key Laboratory for Animal Disease-Resistance Nutrition of China, Ministry of Education, Sichuan Agricultural University, Chengdu, Sichuan, 611130, China; Institute of Animal Nutrition, Key Laboratory for Animal Disease-Resistance Nutrition of China, Ministry of Education, Sichuan Agricultural University, Chengdu, Sichuan, 611130, China; Institute of Animal Nutrition, Key Laboratory for Animal Disease-Resistance Nutrition of China, Ministry of Education, Sichuan Agricultural University, Chengdu, Sichuan, 611130, China; Institute of Animal Nutrition, Key Laboratory for Animal Disease-Resistance Nutrition of China, Ministry of Education, Sichuan Agricultural University, Chengdu, Sichuan, 611130, China; Institute of Animal Nutrition, Key Laboratory for Animal Disease-Resistance Nutrition of China, Ministry of Education, Sichuan Agricultural University, Chengdu, Sichuan, 611130, China; Institute of Animal Nutrition, Key Laboratory for Animal Disease-Resistance Nutrition of China, Ministry of Education, Sichuan Agricultural University, Chengdu, Sichuan, 611130, China; Institute of Animal Nutrition, Key Laboratory for Animal Disease-Resistance Nutrition of China, Ministry of Education, Sichuan Agricultural University, Chengdu, Sichuan, 611130, China; Institute of Animal Nutrition, Key Laboratory for Animal Disease-Resistance Nutrition of China, Ministry of Education, Sichuan Agricultural University, Chengdu, Sichuan, 611130, China; Institute of Animal Nutrition, Key Laboratory for Animal Disease-Resistance Nutrition of China, Ministry of Education, Sichuan Agricultural University, Chengdu, Sichuan, 611130, China; Institute of Animal Nutrition, Key Laboratory for Animal Disease-Resistance Nutrition of China, Ministry of Education, Sichuan Agricultural University, Chengdu, Sichuan, 611130, China

**Keywords:** amino acids, distillers dried grains with solubles, sows, standardized ileal digestibility

## Abstract

This study determined the standardized ileal digestibility (SID) of amino acids (AA) and crude protein (CP) in seven corn distillers dried grains with solubles (DDGS) fed to mid-gestating, late-gestating, and lactating sows. Seven representative corn DDGS samples were collected from ethanol plants in seven different provinces across China. Sixteen sows (Landrace × Yorkshire; parity 4) with a T-cannula in the distal ileum were assigned to a repeated 8 × 3 incomplete Latin square design (three periods and eight diets) during mid-gestation, late-gestation, and lactation stages. The diets included seven experimental diets (DDGS 1–7) and a nitrogen-free diet. Results showed that when fed to sows (mid- and late-gestating and lactating), the ileal digestibility of CP from seven DDGS samples differed significantly (*P *< 0.05). When fed to mid-gestating sows, the SID of AA (excepting His, Lys, Gly, and Pro) showed significant differences (*P *< 0.05). Across all essential AA in DDGS, mean SID value for Leu was the highest (93.57%), mean SID value for Trp was the lowest (78.61%). When fed to late-gestating sows, the SID of AA (excepting Met and Phe) showed significant differences (*P *< 0.05). When fed to lactating sows, only the SID of Trp and Cys for seven DDGS samples showed significant differences (*P *< 0.05). In addition, the SID of CP and most AA in DDGS was significantly higher in mid-gestating sows compared to late-gestating sows, and higher in late-gestating sows compared to lactating sows (*P *< 0.01). Overall, the SID values for CP and most AA (except Gly) in corn DDGS were influenced by sow physiological stage. Our findings provide a reference for the use of corn DDGS in sow diet.

## Teaser

This study provides the standardized ileal digestibility of amino acids from seven sources of corn distillers dried grains with solubles at different physiological stages of sows.

## Introduction

Production of corn distillers dried gains with solubles (DDGS), a major byproduct of organic chemicals and emerging biofuel (ethanol), has increased year upon year (Shad et al., 2021). Corn DDGS, rich in nutrients such as crude protein (CP) and amino acids (AA), is a high-quality substitute for soybean meal (SBM) and corn that has been widely used in pig feed (Han and Liu, 2010). However, the nutrient composition and digestibility of corn DDGS can vary because of differences in raw material quality, origin, fermentation type, and processing method, which affect its use in animal production (Kingsly et al., 2010; Li et al., 2015a). The standardized ileal digestibility (SID) of CP and AA in corn DDGS decreased after oil extraction (Curry et al., 2014; Li et al., 2015b). The SID of lysine in darker-colored corn DDGS was lower (Ganesan et al., 2008; Kingsly et al., 2010); heat also decreased the SID of AA in corn DDGS (Almeida et al., 2013).

Inclusion of corn DDGS in the diet of pregnant and lactating sows can be increased to about 30% without negatively affecting sow or piglet production (Song et al., 2010; Wei et al., 2016). The ileal AA digestibility of common protein sources for sows (e.g., SBM, extruded soybean, and cottonseed meal) varies between sows in different physiological stages (Wang et al., 2023a, 2023b). However, because of difficulties with the installation of ileal cannulas in sows, the SID of AA in DDGS for multiparous sows is unreported. Swine feedstuffs databases are mostly based on the growing pig model (NRC, 2012; CVB Feed Table, 2018). An accurate assessment of the SID of AA in DDGS at different physiological stages of sows is necessary. Accordingly, we report the SID of CP and AA in corn DDGS from different sources in mid- and late-gestating and lactating sows.

## Materials and Methods

All study protocols were reviewed and approved by the Animal Care and Use Committee at Sichuan Agricultural University (SICAU20210038).

### Animals, diets, and experimental design

Twenty-four post-weaned sows (Landrace × Yorkshire; LY) with similar body weights were fitted with a T-cannula at the distal ileum (Stein et al., 1998). T-cannula-fitted sows were allowed to recover for 2 wk after surgery. Estrus synchronization was then performed, and artificial insemination using semen from Duroc boars was conducted on the day of standing heat. At 30 d of gestation, all sows underwent pregnancy diagnosis via a B-mode ultrasonography, from which 16 pregnant sows (parity 4) were subsequently selected. The sixteen sows with a T-cannula in the distal ileum were used to measure SID values for CP and AAs for seven corn DDGS samples. Sows were randomly allocated to a replicated 8 × 3 Youden square design, with 8 diets and 3 periods in each square during mid-gestation (days 48–68), late-gestation (days 88–108), and lactation (days 6–26). Each period lasted 7 d (5 d for adaptation, 2 d for ileal digesta collection). Each treatment was replicated six times. The diets included seven experimental diets formulated with 47% corn DDGS (DDGS 1–7) and a nitrogen-free diet. Seven representative corn DDGS samples were collected from ethanol processing plants in seven different provinces across China. The sources and chemical composition of DDGS are presented in Tables 1 and 2. All diets contained 0.4% chromic oxide as an indigestible marker (Table 3). Basal endogenous CP and AA losses were determined using a nitrogen-free diet method (Adeola et al., 2016). The AA digestibility of DDGS was determined using a direct method, where DDGS was utilized as the sole nitrogen source (Stein et al., 2007).

**Table 1.** Sources, mycotoxin contents and physical properties of corn distillers dried grains with solubles samples used in the experiment (as-fed basis)

NO.^1^	Province	Mycotoxin^1^			Physical property^2^			
		DON (mg/kg)	AFB_1_ (μg/kg)	ZEN (μg/kg)	BD g/L	L*	a*	b*
**1**	Sichuan	1.88	ND^3^	91.33	504	61.27	13.64	41.42
**2**	Liaoning	3.00	ND	332.15	556	64.84	14.56	36.40
**3**	Heilongjiang	2.64	ND	290.00	584	58.89	14.82	37.51
**4**	Xinjiang	ND	5.78	ND	524	56.94	14.62	38.42
**5**	Henan	1.85	10.18	297.45	532	61.63	12.58	38.36
**6**	Jilin	1.38	ND	59.93	484	66.57	14.21	45.85
**7**	Chongqing	1.38	ND	28.43	508	65.67	12.68	45.77

1 DON = deoxynivalenol, AFB_1_ = Aflatoxin B_1_, ZEN = Zearalenone.

2 BD = bulk density, L* = lightness of sample, 0 = black, 100 = white; greater values of a* and b* indicate greater degree of redness and yellowness, respectively.

3 ND = not detected.

**Table 2.** Nutrient composition of 7 corn distillers dried grains with solubles (% dry matter, unless otherwise indicated)^1^

Item^1^	DDGS1	DDGS2	DDGS3	DDGS4	DDGS5	DDGS6	DDGS7	Mean	CV
**DM**	86.95	88.50	88.42	87.83	87.93	87.46	87.67	87.82	0.61
**GE, kcal/kg**	5459.2	4888.9	4912.5	5242.6	5153.9	5301.7	5250.3	5172.7	4.01
**CP**	28.28	29.14	30.52	26.70	30.19	29.17	27.72	28.82	4.70
**EE**	12.13	4.17	3.66	9.50	10.82	12.13	8.84	8.75	40.30
**CF**	6.91	6.89	7.65	7.06	6.91	6.97	7.21	7.09	3.86
**NDF**	37.76	32.32	42.47	36.30	37.62	34.96	39.91	37.33	8.82
**ADF**	13.11	8.79	12.81	10.12	12.82	10.97	12.70	11.62	14.46
**Starch**	3.45	11.86	4.18	5.35	4.21	5.03	5.02	5.59	50.89
**Ca**	0.32	0.19	0.21	0.22	0.20	0.24	0.15	0.22	24.13
**P**	1.00	0.88	1.06	0.88	1.07	0.88	0.96	0.96	8.79
**Ash**	5.01	4.05	4.63	3.98	6.38	4.29	4.59	4.70	17.45
**Indispensable amino acids**									
**Arg**	1.09	1.15	1.29	1.17	1.22	1.19	1.17	1.18	5.23
**His**	0.76	0.76	0.85	0.76	0.79	0.80	0.74	0.78	4.74
**Ile**	0.95	0.99	1.03	0.89	0.99	0.99	0.93	0.97	4.84
**Leu**	3.35	3.66	3.69	3.09	3.51	3.62	3.16	3.44	7.09
**Lys**	0.94	0.85	1.02	0.86	1.02	0.98	0.99	0.95	7.49
**Met**	0.41	0.43	0.41	0.30	0.46	0.41	0.38	0.40	12.58
**Phe**	1.26	1.37	1.44	1.22	1.35	1.38	1.24	1.32	6.27
**Thr**	1.08	1.08	1.17	1.00	1.13	1.10	1.02	1.08	5.46
**Trp**	0.22	0.21	0.23	0.22	0.22	0.21	0.22	0.22	3.16
**Val**	1.26	1.29	1.40	1.22	1.36	1.34	1.22	1.30	5.42
**Dispensable amino acids**									
**Ala**	2.10	2.18	2.22	1.88	2.19	2.19	1.94	2.10	6.48
**Asp**	1.77	1.83	1.95	1.68	1.89	1.83	1.74	1.81	5.04
**Cys**	0.32	0.38	0.39	0.33	0.36	0.37	0.29	0.35	10.40
**Glu**	5.06	5.53	5.52	4.76	5.47	5.53	4.79	5.24	6.82
**Gly**	1.06	1.05	1.19	1.05	1.14	1.10	1.04	1.09	5.19
**Pro**	2.33	2.50	2.63	2.26	2.49	2.50	2.15	2.41	6.94
**Ser**	1.37	1.43	1.53	1.30	1.46	1.44	1.31	1.41	5.94
**Tyr**	1.08	1.12	1.18	1.02	1.14	1.15	1.03	1.10	5.56

1 DDGS = distillers dried grains with solubles, DM = dry matter, GE = gross energy, CP = Crude protein, EE = Ether extract, CF = Crude fiber, NDF = Neutral detergent fiber, ADF = Acid detergent fiber.

**Table 3.** Ingredient composition (as-fed basis) in experimental diets

Ingredient, %	Test diets^1^		N-free diet	
	Gestation	Lactation	Gestation	Lactation
**Cornstarch**	37.10	36.55	69.65	69.05
**Corn DDGS**	47.00	47.00	0.00	0.00
**Soybean oil**	2.00	2.00	2.00	2.00
**Limestone**	1.50	0.75	0.85	0.00
**Dicalcium phosphate**	1.00	2.30	2.20	3.65
**NaCl**	0.40	0.40	0.40	0.40
**Sucrose**	10.00	10.00	10.00	10.00
**Glucose**	0.00	0.00	10.00	10.00
**Solka floc**	0.00	0.00	4.00	4.00
**Chromic oxide**	0.40	0.40	0.40	0.40
**Choline chloride (50%)**	0.20	0.20	0.20	0.20
**Mycotoxin adsorbent**	0.10	0.10	0.00	0.00
**Vitamin premix^2^**	0.10	0.10	0.10	0.10
**Mineral premix^3^**	0.20	0.20	0.20	0.20
**Total**	100.00	100.00	100.00	100.00

1 Test diets were formulated using different corn DDGS sources.

2 Vitamin premix provided per kilogram of complete diet: 6,000 IU Vitamin A; 2,000 IU Vitamin D_3_; 80 IU Vitamin E; 3.8 mg Vitamin K; 2.0 mg Vitamin B_1_; 6.0 mg riboflavin; 4.0 mg Vitamin B_6_; 0.02 mg Vitamin B_12_; 26.0 mg niacin; 18.0 mg pantothenic acid; 3.2 mg folic acid; 0.4 mg biotin;

3 Mineral premix provided per kilogram of complete diet: during gestation - 80 mg iron; 10 mg copper; 100 mg zinc; 25 mg manganese; 0.14 mg iodine; and 0.25 mg selenium. During lactation: 80 mg iron; 20 mg copper; 100 mg zinc; 25 mg manganese; 0.14 mg iodine; and 0.25 mg selenium.

### Feeding, diets, and sample collection

Mid- and late-gestating sows were housed individually in metabolism crates (2.10 × 0.97 × 1.20 m) with smooth sides and plastic covers. Each crate was equipped with a stainless-steel feeder and a nipple drinker. Sows were fed 1.5 kg of feed twice daily (08:00 and 16:00 h), and the room temperature was controlled at 20 ± 2°C. Lactating sows were housed in farrowing crates within a farrowing room and provided with ad libitum access to the experimental diet. The room temperature was controlled at 24 ± 2°C. Nonexperimental sows were fed standard commercial feed. Pigs had free access to water throughout experimentation. Samples of ileal digesta were continuously collected for 12 h (08:00–20:00) on days 6 and 7 of each period following the methodology in Wang et al. (2023b). Ileal digesta treatment prior to analysis was performed in accordance with Wang et al. (2024).

### Chemical analysis and calculations

Sample dry matter (DM; method 930.15), crude ash (method 942.05), CP (method 990.03), EE (method 920.39), AA (method 982.30), and calcium (Ca) and phosphorus (P) (method 985.01) were determined as described by AOAC (2007). Gross energy (GE) content was analyzed using a Parr 6400 automatic oxygen bomb calorimeter (Parr Instrument Co., Moline, IL, USA). Starch content was measured using the polarimetric method described earlier (Jezierny et al., 2017). Neutral detergent fiber (NDF), acid detergent fiber (ADF), and crude fiber (CF) concentrations were determined using a fiber analyzer (Ankom Technology, Macedon, NY, USA; Van Soest et al., 1991).

The zearalenone (ZEN) and deoxynivalenol (DON) analyses were conducted following the procedures outlined in GB/T 23504-2009 and GB/T 23503-2009 standards (Liu et al., 2016; Wu et al., 2016). The analysis of aflatoxin B_1_ (AFB_1_) was conducted using the method described in the previous study (Trucksess et al., 1994). The BD of DDGS was determined as described by Cromwell et al. (2000). Color scores (L*, a*, and b*) of DDGS samples were determined using a Minolta Chroma Meter CR-400 (Minolta, Osaka, Japan). The mean color score was calculated as the average of 10 readings. Lower L* values correspond to darker colors, while higher values indicate lighter colors (0 = black; 100 = white). Higher a* and b* values reflect increased redness and yellowness, respectively.

Basal ileal endogenous CP and AA losses in sows at different physiological stages were determined using the nitrogen-free diet, and the apparent ileal digestibility (AID) and SID values for CP and AAs in corn DDGS were calculated according to Stein et al. (2007).

### Statistical analysis

The normality of residuals and the presence of outliers were assessed using the UNIVARIATE procedure in SAS (SAS Institute Inc., Cary, NC, USA). Data points with mean values differing from treatment means by > 3 × the interquartile range were considered outliers and excluded. Statistical analyses were performed using the PROC MIXED procedure in SAS, with the sow as the experimental unit. The statistical model accounted for the fixed effect of DDGS or diet, as well as the random effects of period, sow, and reproductive phase. To assess differences between pregnancy phases, variance analysis was performed with pregnancy phase as a fixed effect and sow and period as random effects. The LSMEANS statement was used to compute the least squares means for each treatment, and Tukey’s multiple range tests were applied to identify statistical differences. Results were deemed significant at *P *< 0.05, with trends observed at 0.05 ≤ *P *< 0.1.

## Results

### Chemical composition and variability of corn DDGS

As shown in Table 1, DDGS 2 and 3 had relatively higher DON contents (3.00 and 2.64 mg/kg), and DDGS 4 and 5 had higher AFB_1_ contents (5.78 and 10.18 µg/kg). DDGS 2, 5, and 3 had the relatively higher ZEN content (332.15, 297.45, and 290.00 µg/kg). DDGS 6 had the lowest BD. DDGS 6 had the highest L* and b* values, while DDGS 4 had the lowest L*. As shown in Table 2, the DM mean values for CP and EE in the seven DDGS samples were 28.82% (CV 4.70%) and 8.75% (CV 40.30%), respectively. CVs for EE, starch, and Ca exceeded 24%, and those for ADF, Ash, Met, and Cys exceeded 10%.

### Apparent ileal digestibility

The AID of CP and AA in corn DDGS fed to mid-gestating, late-gestating, and lactating sows are shown in Tables 4–6. For mid-gestating sows (Table 4), the AID of CP and most AAs in the seven DDGS types showed significant differences (*P *< 0.05). AID values for CP, Trp, and Ser in DDGS 6 and 7 were lower than for other DDGS. For late-gestating sows (Table 5), AID values for CP and all AAs (excepting Met) in the seven DDGS samples showed significant differences (*P *< 0.05). AID values for CP in DDGS 5 and 6 exceeded those of DDGS 1–3 (*P *< 0.05), and that for DDGS 4 exceeded that for DDGS 1 (*P *< 0.05). AID values for Lys in DDGS 4–7 exceeded those of DDGS 1–3 (*P *< 0.05). The AID value for Thr in DDGS 4–6 exceeded those for DDGS 1 and 3 (*P *< 0.05). For lactating sows (Table 6), the AID of CP, Trp, and Cys in the seven DDGS samples showed significant differences (*P *< 0.05). The AID of CP, Trp, and Cys in DDGS 6 exceeded those for other samples.

**Table 4.** Apparent ileal digestibility of crude protein (CP) and amino acids (AA) in corn DDGS from different origins in mid-gestating sows (%)

Item	1	2	3	4	5	6	7	Mean	SEM	*P*-value
**CP**	75.84^bc^	81.60^a^	79.73^ab^	77.89^ab^	78.45^ab^	71.65^d^	72.43^cd^	76.80	0.70	< 0.01
**Indispensable AA**										
**Arg**	83.62^bc^	86.89^a^	87.66^a^	86.00^ab^	85.30^ab^	80.36^d^	82.02^cd^	84.55	0.52	< 0.01
**His**	84.88	87.52	84.48	85.66	85.15	84.98	84.20	85.27	0.39	0.43
**Ile**	81.88^ab^	85.15^a^	83.57^ab^	82.91^ab^	83.39^ab^	80.64^b^	80.93^ab^	82.64	0.43	0.047
**Leu**	89.94^b^	92.60^a^	90.97^ab^	90.87^ab^	90.60^b^	89.68^b^	89.33^b^	90.57	0.28	0.04
**Lys**	75.53	74.04	75.23	74.95	77.02	74.15	76.40	75.33	0.58	0.84
**Met**	81.59^c^	89.35^a^	89.33^a^	87.46^ab^	84.64^bc^	81.90^c^	84.61^bc^	85.55	0.66	< 0.01
**Phe**	86.26^bc^	89.10^a^	88.55^ab^	86.95^abc^	87.09^abc^	85.52^c^	85.47^c^	86.99	0.34	0.01
**Thr**	74.14^ab^	77.25^a^	77.32^a^	73.39^ab^	74.10^ab^	67.90^c^	71.23^bc^	73.62	0.66	< 0.01
**Trp**	59.53^b^	65.54^ab^	71.50^a^	65.05^ab^	68.56^a^	46.57^c^	51.39^c^	61.16	1.63	< 0.01
**Val**	81.06^bcd^	84.96^a^	84.25^ab^	82.61^abcd^	83.14^abc^	79.67^d^	80.02^cd^	82.25	0.48	< 0.01
**Dispensable AA**										
**Ala**	84.99^cd^	88.17^a^	87.61^ab^	85.58^bcd^	86.38^abc^	83.51^de^	82.70^e^	85.56	0.39	< 0.01
**Asp**	74.00^bc^	80.31^a^	77.78^ab^	75.38^bc^	77.09^ab^	71.31^c^	71.32^c^	75.31	0.68	< 0.01
**Cys**	74.09^ab^	80.75^a^	77.32^a^	81.43^a^	80.10^a^	58.10^c^	64.26^bc^	73.72	1.84	< 0.01
**Glu**	87.74^cd^	90.89^a^	89.71^ab^	88.50^bcd^	89.30^abc^	87.10^d^	86.68^d^	88.56	0.31	< 0.01
**Gly**	59.66	64.72	66.33	63.76	65.27	60.84	56.47	62.44	1.28	0.35
**Pro**	57.95	69.49	69.80	75.87	68.71	55.80	61.58	65.60	2.37	0.20
**Ser**	79.43^c^	84.01^a^	82.63^ab^	80.25^bc^	81.09^abc^	75.79^d^	76.34^d^	79.93	0.56	< 0.01
**Tyr**	85.25^bc^	88.78^a^	87.48^ab^	86.26^bc^	85.89^bc^	84.65^c^	84.69^c^	86.14	0.36	< 0.01

**abcde:** Different superscripts indicate that means in the same row differ (*P *< 0.05).

**Table 5.** Apparent ileal digestibility of crude protein (CP) and amino acids (AA) in corn DDGS from different origins in late-gestating sows (%)

Item	1	2	3	4	5	6	7	Mean	SEM	*P*-value
**CP**	71.30^c^	72.95^bc^	73.19^bc^	77.91^ab^	79.30^a^	80.77^a^	76.99^abc^	76.06	0.83	< 0.01
**Indispensable AA**										
**Arg**	79.06^c^	81.46^c^	81.85^bc^	86.34^a^	85.79^ab^	86.50^a^	87.73^a^	84.11	0.68	< 0.01
**His**	81.57^cd^	82.50^bcd^	80.36^d^	85.76^ab^	84.61^abc^	86.67^a^	84.48^abc^	83.71	0.50	< 0.01
**Ile**	79.10^b^	79.54^b^	79.68^b^	83.38^a^	84.22^a^	83.58^a^	81.95^ab^	81.64	0.52	0.01
**Leu**	87.88^b^	89.64^ab^	88.22^b^	90.80^a^	91.11^a^	90.98^a^	89.46^ab^	89.73	0.31	< 0.01
**Lys**	68.57^b^	64.46^b^	70.00^b^	76.00^a^	75.91^a^	76.45^a^	76.09^a^	72.49	0.95	< 0.01
**Met**	78.72	85.51	75.76	80.35	80.60	80.72	80.22	80.27	0.97	0.24
**Phe**	83.88^b^	85.28^ab^	84.07^b^	86.92^a^	87.20^a^	88.11^a^	86.16^ab^	85.94	0.39	0.01
**Thr**	69.82^c^	70.86^bc^	68.55^c^	75.76^ab^	77.67^a^	76.57^ab^	73.92^abc^	73.31	0.83	< 0.01
**Trp**	49.70^cd^	50.77^bcd^	48.49^d^	64.68^a^	59.16^ab^	58.00^abc^	59.15^ab^	55.71	1.36	< 0.01
**Val**	76.69^b^	78.86^ab^	76.93^b^	82.24^a^	81.48^a^	81.39^a^	80.87^a^	79.78	0.54	0.01
**Dispensable AA**										
**Ala**	82.06^d^	83.98^bcd^	82.74^cd^	85.97^abc^	86.66^ab^	87.65^a^	84.53^abcd^	84.80	0.47	< 0.01
**Asp**	70.10^b^	73.29^b^	70.09^ab^	76.68^a^	78.27^a^	77.73^a^	75.55^ab^	74.53	0.84	0.01
**Cys**	70.17^bc^	73.86^abc^	66.68^c^	81.81^a^	82.48^a^	83.12^a^	76.98^ab^	76.44	1.46	< 0.01
**Glu**	85.45^c^	86.72^bc^	85.45^c^	89.04^ab^	89.67^a^	90.42^a^	87.77^abc^	87.79	0.44	< 0.01
**Gly**	48.35^c^	51.61^bc^	57.42^abc^	62.17^ab^	69.76^a^	67.19^a^	63.24^ab^	59.96	1.90	< 0.01
**Pro**	48.30^c^	51.46^c^	55.33^bc^	73.05^a^	78.93^a^	75.85^a^	68.52^ab^	64.49	2.46	< 0.01
**Ser**	75.36^b^	78.39^ab^	75.28^b^	81.68^a^	82.87^a^	82.48^a^	79.96^a^	79.43	0.69	< 0.01
**Tyr**	84.68^b^	84.38^b^	83.73^b^	86.65^ab^	87.78^a^	88.34^a^	86.22^ab^	85.97	0.42	0.01

abcd Different superscripts indicate that means in the same row differ (*P *< 0.05).

**Table 6.** Apparent ileal digestibility of crude protein (CP) and amino acids (AA) in corn DDGS from different origins in lactating sows (%)

Item	1	2	3	4	5	6	7	Mean	SEM	*P*-value
**CP**	72.51^bc^	72.57^bc^	70.52^bc^	69.46^c^	75.32^ab^	77.33^a^	72.23^bc^	72.85	0.66	0.02
**Indispensable AA**										
**Arg**	80.88	82.27	80.33	80.18	79.19	86.11	79.71	81.24	0.73	0.19
**His**	82.33	82.14	80.82	79.69	76.29	85.09	78.84	80.74	0.80	0.07
**Ile**	81.24	81.98	80.81	78.74	78.26	83.98	77.38	80.34	0.70	0.17
**Leu**	89.55	90.28	89.65	87.63	87.02	90.66	86.54	88.76	0.48	0.12
**Lys**	71.39	66.64	69.11	64.76	64.66	74.31	66.05	68.13	1.18	0.19
**Met**	88.52	88.18	87.26	84.28	88.07	91.84	86.51	87.81	0.66	0.07
**Phe**	85.88	86.73	85.65	83.98	84.15	87.94	83.29	85.37	0.52	0.23
**Thr**	70.15	68.87	66.75	65.50	66.26	72.74	63.93	67.74	0.97	0.24
**Trp**	52.46^bcd^	56.74^abc^	48.87^cd^	52.71^bcd^	58.65^ab^	62.65^a^	47.30^d^	54.20	1.28	0.01
**Val**	80.32	80.65	79.67	77.36	78.02	81.79	74.84	78.95	0.63	0.07
**Dispensable AA**										
**Ala**	83.14	84.58	81.24	79.83	80.32	85.67	79.87	82.09	0.66	0.09
**Asp**	71.11	73.73	69.96	68.44	69.82	76.41	65.53	70.71	1.01	0.14
**Cys**	74.15^b^	87.51^a^	71.35^b^	84.47^a^	91.19^a^	89.80^a^	75.37^b^	81.98	1.52	< 0.01
**Glu**	86.84	87.29	86.36	84.18	85.68	88.47	83.58	86.06	0.49	0.09
**Gly**	58.02	52.74	49.60	54.31	62.09	66.47	51.38	56.37	1.88	0.37
**Pro**	68.99	63.50	61.81	56.93	65.20	69.77	55.50	63.10	2.03	0.67
**Ser**	77.53	78.21	76.19	75.06	75.67	80.96	72.87	76.64	0.77	0.18
**Tyr**	84.93	87.62	83.29	84.94	85.76	89.11	84.03	85.67	0.57	0.10

abcd Different superscripts indicate that means in the same row differ (*P *< 0.05).

A comparison of AID for the seven DDGS samples in sows at different physiological stages is presented in Table 7. Compared with mid- and late-gestating sows, lactating sows had significantly lower AID values for CP, Arg, His, Lys, Thr, Ala, Asp, Glu, and Ser (*P *< 0.05). Compared with mid-gestating sows, lactating sows had lower AID values for Ile, Leu, Phe, Trp, Val, and Gly (*P *< 0.05). Compared with gestating sows, lactating sows had significantly higher AID values for Met and Cys (*P *< 0.05). Compared with late-gestating sows, mid-gestating sows had greater AID values for Lys, Met, Thr, Trp, and Val (*P *< 0.05).

**Table 7.** Comparison of ileal digestibility values for crude protein (CP) and amino acids (AA) in corn DDGS from different origins in sows

Item, %	AID			SEM	*P*-value	SID			SEM	*P*-value
	Mid- gestation	Late- gestation	Lactation			Mid-gestation	Late-gestation	Lactation		
**CP**	76.73^a^	76.06^a^	72.77^b^	0.45	< 0.01	86.22^a^	84.26^a^	80.03^b^	0.49	< 0.01
**Indispensable AA**										
**Arg**	84.43^a^	84.08^a^	81.12^b^	0.39	< 0.01	91.71^a^	89.40^b^	85.52^c^	0.44	< 0.01
**His**	85.18^a^	83.71^a^	80.65^b^	0.38	< 0.01	89.77^a^	87.31^b^	83.97^c^	0.40	< 0.01
**Ile**	82.66^a^	81.57^ab^	80.30^b^	0.32	0.01	88.71^a^	86.69^b^	84.75^c^	0.36	< 0.01
**Leu**	90.60^a^	89.66^ab^	88.72^b^	0.22	< 0.01	93.57^a^	91.73^b^	91.06^b^	0.23	< 0.01
**Lys**	75.26^a^	72.51^b^	68.19^c^	0.58	< 0.01	82.67^a^	77.80^b^	72.81^c^	0.63	< 0.01
**Met**	85.41^b^	80.30^c^	87.73^a^	0.54	< 0.01	90.44^b^	88.52^b^	93.17^a^	0.44	< 0.01
**Phe**	87.03^a^	85.89^ab^	85.34^b^	0.25	0.02	91.45^a^	89.24^b^	88.41^b^	0.27	< 0.01
**Thr**	73.72^a^	73.20^b^	67.78^c^	0.53	< 0.01	84.57^a^	79.89^b^	76.04^c^	0.58	< 0.01
**Trp**	60.96^a^	55.71^b^	54.29^b^	0.86	< 0.01	78.69^a^	68.64^b^	67.52^b^	0.93	< 0.01
**Val**	82.17^a^	79.74^b^	79.01^b^	0.34	< 0.01	88.61^a^	84.03^b^	83.53^b^	0.38	< 0.01
**Dispensable AA**										
**Ala**	85.59^a^	84.73^a^	82.04^b^	0.33	< 0.01	91.21^a^	88.84^b^	85.60^c^	0.37	< 0.01
**Asp**	75.08^a^	74.53^a^	70.70^b^	0.51	< 0.01	84.10^a^	80.92^b^	76.81^c^	0.56	< 0.01
**Cys**	74.01^b^	76.14^b^	81.97^a^	0.98	< 0.01	80.67^ab^	78.50^b^	84.78^a^	0.92	0.02
**Glu**	88.52^a^	87.72^a^	86.02^b^	0.26	< 0.01	92.42^a^	90.39^b^	88.71^c^	0.28	< 0.01
**Gly**	62.26^a^	60.09^ab^	56.64^b^	1.13	0.04	80.45	77.12	78.81	1.02	0.39
**Pro**	65.19	64.24	62.92	1.51	0.30	91.59^a^	91.51^a^	74.01^b^	1.58	< 0.01
**Ser**	79.94^a^	79.43^a^	76.66^b^	0.41	< 0.01	87.95^a^	85.07^b^	82.57^c^	0.44	< 0.01
**Tyr**	86.15	85.91	85.62	0.26	0.72	90.80^a^	89.42^b^	88.50^b^	0.27	< 0.01

abc Means within a row with different lowercase superscripts for AID and SID indicate significant differences (*P *< 0.05).

### Endogenous AA losses in sows

AAs with the highest contents in endogenous protein from the sows’ ileum were Pro, Gly, Glu, Asp, Ala, Ser, and Thr (Table 8). Basal ileal endogenous losses (BEL) for essential AAs for mid-gestating sows ranged 122.36 mg/kg dry matter intake (DMI) for Met to 554.38 mg/kg DMI for Thr; for late-gestating sows these ranged 115.27 mg/kg DMI for Trp to 326.47 mg/kg DMI for Thr; and for lactating sows, 117.32 mg/kg DMI for His to 409.41 mg/kg DMI for Thr. BEL for most AAs (excepting Ile, Met, Phe, Thr, Trp, and Gly) showed significant differences across sow physiological stages. BEL values for Arg, Val, Ala, Asp, Cys, Glu, Ser, and Tyr in mid-gestating sows exceeded those for late-gestating and lactating sows (*P *< 0.05). BEL values for His and Lys in mid-gestating sows exceeded those of lactating sows (*P *< 0.05). The BEL value for Leu in mid-gestating sows exceeded that of late-gestating sows (*P *< 0.05), and that for Pro in gestating sows exceeded that for lactating sows (*P *< 0.05).

**Table 8.** Basal ileal endogenous losses (mg/kg dry matter intake) of crude protein (CP) and amino acid (AA) in sows

Item	Mid-gestating sows	Late-gestating sows	Lactating sows	SEM	*P*-value
**CP**	13361.84	11033.58	9759.26	739.75	0.11
**Indispensable AA**					
**Arg**	433.08^a^	293.42^b^	247.68^b^	31.33	0.02
**His**	173.32^a^	130.53^ab^	117.32^b^	10.16	0.04
**Ile**	287.25	227.16	196.66	17.11	0.07
**Leu**	495.20^a^	320.99^b^	360.48^ab^	31.21	< 0.01
**Lys**	313.57^a^	240.70^ab^	201.92^b^	19.29	0.03
**Met**	122.36	149.73	127.01	13.95	0.74
**Phe**	293.75	211.86	188.84	19.74	0.05
**Thr**	554.38	326.47	409.41	40.86	0.06
**Trp**	165.59	115.27	118.09	10.16	0.05
**Val**	399.40^a^	264.64^b^	267.04^b^	24.44	0.02
**Dispensable AA**					
**Ala**	564.28^a^	401.53^b^	343.05^b^	34.55	0.01
**Asp**	767.39^a^	529.31^b^	503.17^b^	46.61	0.02
**Cys**	152.26^a^	30.40^b^	65.29^b^	15.70	< 0.01
**Glu**	978.19^a^	646.11^b^	642.06^b^	60.87	0.02
**Gly**	1006.08	849.65	1131.56	105.81	0.62
**Pro**	2983.89^a^	3122.62^a^	1291.77^b^	338.96	0.04
**Ser**	533.64^a^	356.83^b^	379.37^b^	33.49	< 0.01
**Tyr**	265.13^a^	183.13^b^	152.11^b^	17.37	0.01

ab Different superscripts indicate that means in the same row differ (*P *< 0.05).

### Standardized ileal digestibility

For mid-gestating sows (Table 9), the SID of CP and most AAs (excepting His, Lys, Gly, and Pro) in DDGS samples differed significantly (*P *< 0.05). The SID of CP, Arg, and Trp in DDGS 1–5 exceeded that for DDGS 6 and 7 (*P *< 0.01). SID values for all AAs (excepting His, Lys, Gly, and Pro) in DDGS 2 exceeded those for DDGS 6 and 7.

**Table 9.** Standardized ileal digestibility of crude protein (CP) and amino acids (AA) in corn DDGS from different origins in mid-gestating sows (%)

Item	1	2	3	4	5	6	7	Mean	SEM	*P*-value
**CP**	85.92^b^	91.18^a^	88.80^ab^	88.14^ab^	87.73^ab^	81.32^c^	80.91^c^	86.29	0.75	< 0.01
**Indispensable AA**										
**Arg**	91.81^a^	94.52^a^	94.48^a^	93.59^a^	92.65^a^	87.67^b^	88.11^b^	91.83	0.57	< 0.01
**His**	89.79	92.31	88.86	90.61	90.17	89.36	88.08	89.88	0.43	0.21
**Ile**	88.34^abc^	91.40^a^	89.42^ab^	89.83^ab^	89.90^ab^	86.69^bc^	85.61^c^	88.74	0.49	0.01
**Leu**	93.18^bc^	95.52^a^	93.89^ab^	94.38^ab^	93.83^ab^	92.57^bc^	91.60^c^	93.57	0.31	0.01
**Lys**	83.20	83.01	82.47	83.36	84.36	81.26	81.31	82.71	0.59	0.84
**Met**	87.23^c^	93.81^a^	93.96^a^	93.35^ab^	89.71^bc^	87.02^c^	89.08^c^	90.59	0.65	< 0.01
**Phe**	91.09^abc^	93.58^a^	92.81^a^	91.98^ab^	91.87^ab^	89.93^bc^	89.12^c^	91.48	0.37	< 0.01
**Thr**	85.04^ab^	88.35^a^	87.45^a^	85.24^ab^	85.31^ab^	78.35^c^	81.31^bc^	84.43	0.69	< 0.01
**Trp**	78.46^a^	84.44^a^	84.01^a^	83.89^a^	85.26^a^	65.38^b^	68.85^b^	78.61	1.62	< 0.01
**Val**	87.98^ab^	91.57^a^	90.33^a^	89.55^a^	89.91^a^	86.02^b^	85.42^b^	88.68	0.53	< 0.01
**Dispensable AA**										
**Ala**	90.79^bc^	93.67^a^	93.02^ab^	91.95^ab^	92.20^ab^	88.93^cd^	87.63^d^	91.17	0.45	< 0.01
**Asp**	83.25^bc^	89.37^a^	86.17^ab^	85.05^b^	86.07^ab^	78.29^d^	80.47^cd^	84.09	0.69	< 0.01
**Cys**	80.48^ab^	86.28^a^	82.78^ab^	87.43^a^	86.58^a^	67.29^c^	72.11^bc^	80.42	1.74	< 0.01
**Glu**	91.87^bcd^	94.69^a^	93.46^ab^	92.91^abc^	93.33^ab^	90.75^cd^	90.09^d^	92.44	0.35	< 0.01
**Gly**	79.44	85.06	84.05	83.86	84.66	75.71	72.47	80.75	1.50	0.13
**Pro**	86.45	88.59	98.01	105.66	97.27	81.85	83.57	91.63	2.57	0.11
**Ser**	87.75^b^	91.90^a^	90.05^ab^	88.92^ab^	89.14^ab^	83.01^c^	84.75^c^	87.93	0.57	< 0.01
**Tyr**	90.20^bc^	93.31^a^	92.07^ab^	91.6^ab^	90.82^abc^	89.31^bc^	88.43^c^	90.83	0.39	< 0.01

abcd Different superscripts indicate that means in the same row differ (*P *< 0.05).

For late-gestating sows (Table 10), the SID of CP and most AAs (excepting Met and Phe) in DDGS samples differed (*P *< 0.05). The SID of CP in DDGS 5 and 6 exceeded that for DDGS 1–3 (*P *< 0.01). SID values for Lys in DDGS 4–7 exceeded those for DDGS 1–3. SID values for all AAs (excepting Met, Phe, Trp, and Gly) in DDGS 6 exceeded those for DDGS 1 and 3. For lactating sows (Table 11), SID values for all AAs (excepting Trp and Cys) in DDGS samples did not differ significantly. The SID of CP in DDGS 6 exceeded those for DDGS 2, 3, and 4 (*P *< 0.05).

**Table 10.** Standardized ileal digestibility of crude protein (CP) and amino acids (AA) in corn DDGS from different origins in late-gestating sows (%)

Item	1	2	3	4	5	6	7	Mean	SEM	*P*-value
**CP**	79.72^c^	81.07^bc^	80.97^bc^	86.45^ab^	87.03^a^	88.83^a^	85.76^ab^	84.26	0.73	< 0.01
**Indispensable AA**										
**Arg**	84.89^c^	86.78^bc^	87.01^bc^	91.37^a^	90.94^ab^	91.85^a^	93.14^a^	89.43	0.67	< 0.01
**His**	85.24^cd^	85.98^bcd^	83.89^d^	89.30^ab^	88.28^abc^	90.18^a^	88.30^abc^	87.31	0.51	< 0.01
**Ile**	84.17^b^	84.66^b^	84.64^b^	88.63^a^	89.32^a^	88.67^a^	87.18^ab^	86.75	0.52	0.01
**Leu**	90.01^b^	91.53^ab^	90.23^b^	92.98^a^	93.13^a^	92.99^a^	91.65^ab^	91.79	0.31	< 0.01
**Lys**	74.36^bc^	69.74^c^	75.37^b^	81.72^a^	81.40^a^	82.07^a^	81.27^a^	77.99	0.96	< 0.01
**Met**	88.24	91.57	86.32	89.13	89.01	86.52	88.59	88.48	0.83	0.70
**Phe**	87.51	88.57	87.45	90.48	90.67	90.14	89.84	89.24	0.41	0.11
**Thr**	76.54^c^	77.30^bc^	75.18^c^	82.62^ab^	84.17^a^	83.34^a^	80.80^abc^	79.99	0.84	< 0.01
**Trp**	62.89^bc^	63.95^bc^	61.64^c^	76.34^a^	72.24^ab^	71.07^ab^	72.33^ab^	68.64	1.33	< 0.01
**Val**	81.14^b^	83.00^ab^	81.19^b^	86.46^a^	85.73^a^	85.77^a^	85.20^a^	84.07	0.54	< 0.01
**Dispensable AA**										
**Ala**	86.23^c^	87.79^bc^	86.81^c^	90.30^ab^	90.67^ab^	91.75^a^	88.85^abc^	88.91	0.48	< 0.01
**Asp**	76.67^b^	79.43^ab^	76.43^b^	83.23^a^	84.38^a^	84.20^a^	82.08^ab^	80.92	0.84	0.01
**Cys**	72.80^bc^	75.88^abc^	68.79^c^	83.92^a^	84.94^a^	85.86^a^	79.48^ab^	78.81	1.47	< 0.01
**Glu**	88.21^c^	89.20^bc^	88.10^c^	91.81^ab^	92.22^a^	93.03^a^	90.60^abc^	90.45	0.43	< 0.01
**Gly**	65.78^c^	68.59^bc^	73.94^abc^	79.01^abc^	86.26^a^	84.55^a^	80.65^ab^	76.97	1.89	0.01
**Pro**	76.18^b^	77.24^b^	81.36^b^	100.15^a^	105.59^a^	103.34^a^	98.13^a^	91.71	2.50	< 0.01
**Ser**	81.27^b^	83.66^ab^	80.85^b^	87.48^a^	88.29^a^	88.12^a^	85.84^a^	85.07	0.69	< 0.01
**Tyr**	88.12^bc^	87.96^bc^	87.22^c^	90.27^ab^	91.16^a^	91.66^a^	89.89^abc^	89.47	0.41	0.01

abcd Different superscripts indicate that means in the same row differ (*P* < 0.05).

**Table 11.** Standardized ileal digestibility of crude protein (CP) and amino acids (AA) in corn DDGS from different origins in lactating sows (%)

Item	1	2	3	4	5	6	7	Mean	SEM	*P*-value
**CP**	79.95^ab^	79.59^b^	77.43^b^	77.22^b^	82.11^ab^	84.62^a^	79.89^ab^	80.11	0.65	0.03
**Indispensable AA**										
**Arg**	85.35	86.86	84.20	84.47	83.63	90.55	84.51	85.65	0.74	0.18
**His**	85.48	85.54	83.71	83.09	79.68	88.48	82.55	84.08	0.80	0.07
**Ile**	85.43	86.40	84.78	83.54	82.60	88.51	82.34	84.80	0.69	0.21
**Leu**	91.77	92.45	91.73	90.21	89.33	93.00	89.22	91.10	0.47	0.19
**Lys**	75.96	72.36	70.82	70.41	69.58	79.48	71.28	72.84	1.17	0.21
**Met**	93.43	93.34	92.35	90.34	93.15	97.16	93.15	93.27	0.63	0.14
**Phe**	88.87	89.67	88.39	87.28	87.10	91.04	86.81	88.45	0.51	0.30
**Thr**	78.04	77.21	74.21	74.35	74.13	81.21	73.11	76.04	0.96	0.28
**Trp**	65.91^bc^	70.22^ab^	62.34^bc^	66.20^bc^	70.56^ab^	76.11^a^	60.73^c^	67.44	1.27	0.02
**Val**	84.51	85.21	83.60	82.10	82.62	86.39	79.97	83.49	0.61	0.12
**Dispensable AA**										
**Ala**	86.50	88.03	84.51	83.78	83.79	89.27	83.88	85.68	0.65	0.12
**Asp**	77.02	79.81	75.46	75.02	75.66	82.69	72.28	76.85	1.00	0.16
**Cys**	78.02^b^	89.99^a^	74.88^b^	86.98^a^	92.96^a^	92.34^a^	78.23^b^	84.77	1.43	< 0.01
**Glu**	89.42	89.84	88.79	87.10	88.27	91.14	86.62	88.74	0.48	0.16
**Gly**	79.69	76.48	69.60	77.30	83.07	86.28	76.05	78.35	1.88	0.37
**Pro**	80.85	82.15	72.48	65.71	76.93	74.78	69.62	74.65	2.08	0.41
**Ser**	83.26	83.98	81.49	81.41	81.33	86.97	79.52	82.57	0.76	0.23
**Tyr**	87.79	90.39	86.07	88.01	88.37	92.01	87.22	88.55	0.57	0.11

abc Different superscripts indicate that means in the same row differ (*P *< 0.05).

SID values for the seven DDGS types in sows at different physiological stages are detailed in Table 7. Compared with gestating sows, lactating sows had higher SID values for Met and lower SID values for CP and Pro (*P *< 0.01). Lactating sows had greater SID values for Cys compared with late-gestating sows (*P *< 0.05). Compared with lactating sows, late-gestating sows had higher SID values for Arg, His, Ile, Lys, Thr, Ala, Asp, Glu, and Ser, but these values were lower than those for mid-gestating sows (*P *< 0.01). Mid-gestating sows had higher SID values for Leu, Phe, Trp, Val, and Tyr compared with late-gestating and lactating sows (*P *< 0.01).

## Discussion

The CP, CF, NDF, and ADF contents of each DDGS sample were within previously reported ranges (NRC, 2012; Espinosa et al., 2019). The Ca and P contents in all DDGS samples generally exceeded NRC (2012) recommendations; higher Ca and P contents in DDGS 1 may be related to levels of DDS re-drying during processing (Kingsly et al., 2010; Iram et al., 2022). With increased proportion of DDS, the BD and EE also increased (Kingsly et al., 2010). Our 7 corn DDGS samples were collected from different factories, and samples with lower EE content tended to have higher BD. This suggests that the finer the corn DDGS particles, the higher the BD, and the better the oil-extraction efficiency (Han and Liu, 2010; Liu, 2011). The average Lys content in corn DDGS samples varies over time: for 118 samples in 2002, it was 0.85% (CV 17.3%); for 10 samples in 2006, it was 0.79% (CV 7.7%) (Spiehs et al., 2002; Stein et al., 2006). Lys is a limiting AA, and its content is significantly affected by processing temperature (Liu, 2011). We report an average Lys content for 7 corn DDGS samples to have increased, indicating progress in production technology.

Few studies have reported the AID and SID of CP and AA in DDGS for gestating and lactating sows. We report significant differences in them among seven DDGS samples, which are consistent with previous findings reported in growing pigs (Li et al., 2015b; Espinosa et al., 2019). It is reported that the processing method and drying of oil-extracted and non-oil-extracted corn DDGS most affect CP and AA digestibility of corn DDGS (Li et al., 2015b). A previous study on growing pigs indicated that conventional DDGS (11.5% fat) has greater SID values for most AA compared with DDGS that contains less fat (7.5% and 6.9% fat) and inclusion of additional oil to diets containing low-fat DDGS does not increase AID or SID of AA, because the AA components in corn DDGS have already undergone the Maillard reaction (Curry et al., 2014).

DDGS 2 had the lowest relative Lys content and relatively higher starch content, indicating that the ethanol extraction was not as complete in this sample (plus the extra starch would dilute the other chemical contents like EE). DDGS 2 had low CF, NDF, and ADF contents, and a lighter color (with a relatively smaller b* value). Changes in corn DDGS color during production are mainly attributed to the Maillard reaction caused by late-stage heating and increased proportion of DDS (Han and Liu, 2010; Urriola et al., 2013). It is reported that lighter-colored DDGS sources may have higher analyzed lysine content and greater digestibility of lysine and AAs (Fastinger and Mahan, 2006).

Average CP and AA digestibility of the seven corn DDGS samples in sows exceeded the recommended values for growing pigs provided by NRC (2012). Compared with the SID of CP in NRC (2012) (high-oil corn DDGS, EE > 10%), we report a SID 12.29% higher in mid-gestation, 10.26% higher in late-gestation, and 6.11% higher in lactation stages. In lactating sows, the SID-CP was 9.96%, 2.21%, 3.15%, and 0.01% higher than values reported by Stein et al. (2006, 10 corn DDGS samples), Almeida et al. (2013, 1 unheated corn DDGS sample), Li et al. (2015b, 2 full-oil DDGS digestibility data), and Espinosa et al. (2019, 9 low-oil corn DDGS samples). The SID-Lys in lactating sows was 18.65, 6.93, 5.86, and 8.73% higher than corresponding values in these publications. The CP and AA digestibility of corn DDGS differs significantly across mid-, and late-gestation, and lactation stages. This is consistent with our team’s previous findings on the SID AA of SBM and full-fat soybean in sows at different physiological stages (Wang et al., 2023a, 2023b). In this study, digestibility is highest in mid-gestation, followed by late-gestation, then lactation. It has been reported that ileal endogenous losses of CP and AA are related to pig dry matter intake, and that restricting feed intake during pregnancy affects the SID of AA (Stein et al., 1999; Stein et al., 2001). However, previous study by our research group has shown that: feeding level did not affect the SID AA in sows (Wang et al., 2023c).

In addition to nutritional composition and processing techniques, the digestibility of CP and AA in corn DDGS may also be influenced by mycotoxin contamination. The most common mycotoxins in corn DDGS are DON, AFB_1_, and ZEN, with DON primarily affecting feed intake in pigs (Kingsly et al., 2010; Liu, 2011). Long-term feeding of corn DDGS may reduce the reproductive performance of sows, possibly because mycotoxin contamination or excessive heating leads to fat peroxidation (Li et al. 2014; Liu et al., 2018). DDGS samples 2 and 3, containing the highest levels of DON and ZEN, exhibited the highest initial digestibility, which gradually decrease during late gestation and lactation. This indicates that the cumulative effects of mycotoxins may have a negative impact on AA digestibility.

## Conclusions

We report the SID of CP and AA in corn DDGS obtained from different sources when fed to sows in mid- and late-gestation, and lactation stages. Significant variability exists in the nutritional composition and AA digestibility of corn DDGS from different sources. Long-term feeding of corn DDGS to sows can affect AA digestibility because of its mycotoxin content. Additionally, the SID of CP and all AA (excepting Gly) in corn DDGS is influenced by the sow’s physiological stage. These results represent a comprehensive reference for the accurate use of DDGS in the diets of pregnant and lactating sows.

## Abbreviations

DDGS: distillers dried gains with solubles
SID: standardized ileal digestibility
AA: amino acids
CP: crude protein
SBM: soybean meal
EE: ether extract
BD: bulk density
DM: dry matter
Ca: calcium
P: phosphorus
GE: gross energy
NDF: neutral detergent fiber
ADF: acid detergent fiber
CF: crude fiber
ZEN: zearalenone
DON: deoxynivalenol
AFB1: aflatoxin B_1_
AID: apparent ileal digestibility
BEL: basal ileal endogenous losses
DMI: dry matter intake
DDS: dried distillers solubles
